# Safety and effectiveness of neoadjuvant PD-1 inhibitor (toripalimab) plus chemotherapy in stage II–III NSCLC (LungMate 002): an open-label, single-arm, phase 2 trial

**DOI:** 10.1186/s12916-022-02696-4

**Published:** 2022-12-30

**Authors:** Xinsheng Zhu, Liangdong Sun, Nan Song, Wenxin He, Boxiong Xie, Junjie Hu, Jing Zhang, Jie Yang, Jie Dai, Dongliang Bian, Haoran Xia, Fenghuan Sun, Anwen Xiong, Jie Luo, Lele Zhang, Huansha Yu, Ming Liu, Hongcheng Liu, Haifeng Wang, Haiping Zhang, Chang Chen, Chunyan Wu, Liang Duan, Yuming Zhu, Peng Zhang, Gening Jiang

**Affiliations:** 1 Department of Thoracic Surgery, Shanghai Pulmonary Hospital, School of Medicine, Tongji University, Shanghai, 200092 China; 2 Department of Oncology, Shanghai Pulmonary Hospital, School of Medicine, Tongji University, Shanghai, 200092 China; 3 Central Laboratory, Shanghai Pulmonary Hospital, School of Medicine, Tongji University, Shanghai, 200092 China; 4 Department of Pathology, Shanghai Pulmonary Hospital, School of Medicine, Tongji University, Shanghai, 200092 China; 5 Department of Thoracic Surgery, Shanghai Pulmonary Hospital, School of Medicine, Tongji University, No. 507 Zhengmin Road, Shanghai, 200433 China

**Keywords:** Neoadjuvant chemoimmunotherapy, Non-small-cell lung cancer, Potentially resectable disease, Transcriptomic characteristics

## Abstract

**Background:**

This trial aimed to analyse the safety, effectiveness and transcriptomic characteristics of neoadjuvant toripalimab plus chemotherapy in II–III non-small-cell lung cancer (NSCLC).

**Methods:**

Patient eligibility mainly involved treatment-naive, clinical stage II–III and wild-type EGFR/ALK NSCLC. The patients received 2–4 cycles of toripalimab (240 mg q3w) plus carboplatin-based chemotherapy. After the second treatment cycle, all patients were re-evaluated by a multidisciplinary team. Candidates eligible for surgery underwent surgery; otherwise, patients received the remaining treatment cycles. The primary endpoints were safety and major pathological response (MPR). Secondary endpoints were R0 resection rate, progression-free survival (PFS) and overall survival (OS). RNA sequencing of baseline and post-treatment samples was conducted to explore the transcriptomic characteristics of the therapeutic response.

**Results:**

In total, 50 eligible patients were enrolled, including 12 (24.0%) with resectable disease (RD) and 38 (76.0%) with potentially resectable disease (PRD). Treatment-related adverse events (TRAEs) were recorded in 48 cases (96.0%). Severe TRAEs occurred in 3 (6.0%) cases, including myelosuppression, drug-induced liver injury and death related to haemoptysis. The objective response rate (ORR) was 76.0%, with 8 (16.0%) patients having a complete response (CR), 30 (60.0%) partial response (PR), 10 (20.0%) stable disease (SD) and 2 (4.0%) progressive disease (PD). Surgery could be achieved in 12 (100%) patients with RD and 25 (65.8%) with PRD; 1 (2.0%) with PRD refused surgery. Therefore, R0 resection was performed for all 36 (100%) patients who underwent surgery; 20 (55.6%) achieved MPR, including 10 (27.8%) with a complete pathological response (pCR). The *CHI3L1* (chitinase-3-like protein 1) immunohistochemistry (IHC) expression of baseline tumour samples could predict the therapeutic response (AUC=0.732), OS (*P*=0.017) and PFS (*P*=0.001). Increased PD-1 expression, T cell abundance and immune-related pathway enrichment were observed in post-treatment samples compared to baseline in the response group (CR+PR) but not in the non-response group (SD+PD).

**Conclusions:**

Neoadjuvant toripalimab plus chemotherapy was safe and effective, with a high MPR and manageable TRAEs for II–III NSCLC, even converting initially PRD to RD. Disparate transcriptomic characteristics of therapeutic efficiency were observed, and *CHI3L1* expression predicted therapeutic response and survival.

**Trial registration:**

ChiCTR1900024014, June 22, 2019.

**Supplementary Information:**

The online version contains supplementary material available at 10.1186/s12916-022-02696-4.

## Background

The prognosis of stage II–III non-small-cell lung cancer (NSCLC) remains poor, and multimodality therapies have been considered as the standard treatment regimen over the past few decades [1–3]. However, the value of chemoradiotherapy and targeted therapy is reported to be limited, with only a mild increase in survival [4, 5]. Programmed cell death receptor 1 (PD-1) and programmed cell death ligand 1 (PD-L1) checkpoint inhibitors have promising antitumour effects, and their value in neoadjuvant chemoimmunotherapy has been proven in resectable IB-IIIA NSCLC, with substantial improvement in pathological response [6–8].

Neoadjuvant chemoimmunotherapy can also downstage initially unresectable NSCLC, converting into resectable disease (RD) and significantly improving survival in patients who receive surgery compared with those who do not [9], which should be further validated in prospective trials. In addition, not all patients benefit from neoadjuvant immunotherapy, and predictors remain unclear [10, 11]. Integrative insight into the intrinsic and extrinsic molecular characteristics of patients that affect the immunotherapy response is critically needed.

Therefore, we initiated this trial to characterize the safety and effectiveness of neoadjuvant toripalimab, a humanized PD-1 inhibitor [12], plus chemotherapy in patients with stage II–III NSCLC. We also analysed the feasibility of neoadjuvant chemoimmunotherapy for potentially resectable disease (PRD) and used RNA sequencing of baseline and post-treatment samples to explore the transcriptomic signatures associated with the therapeutic efficiency.

## Methods

### Design and participants

LungMate 002 was an investigator-initiated, open-label, single-arm, phase 2 clinical trial at Shanghai Pulmonary Hospital that was carried out in accordance with the Declaration of Helsinki. The study protocol was approved by the Ethics Committee of Shanghai Pulmonary Hospital (19216XW-4). Written informed consent was obtained from all patients before enrolment. The main inclusion criteria were as follows: (1) age 18–80 years, (2) Eastern Cooperative Oncology Group (ECOG) performance status 0–1, (3) clinical stage II–III (American Joint Committee on Cancer 8th edition criteria), (4) wild-type EGFR (epidermal growth factor receptor) and ALK (anaplastic lymphoma kinase), (5) histologically or cytologically diagnosed NSCLC and (6) adequate organ function. The main exclusion criteria of the study were as follows: (1) previous treatment with any antitumour drug, (2) concurrent additional malignancy or previous malignant tumour within 5 years, (3) active autoimmune diseases treated with systemic corticosteroid or other immunosuppressive therapy and (4) symptomatic grade 3 or 4 interstitial lung disease. The full details of the inclusion and exclusion criteria are provided in Additional file 1: Table S1.

The clinical stage was diagnosed by computed tomography (CT) and positron emission tomography (PET). Histologic diagnosis was obtained by percutaneous needle biopsy or endobronchial ultrasound biopsy. The lymph node status was evaluated radiologically and/or pathologically (radiological positive node was defined as a diameter larger than 1.5 cm on CT or indicated by PET-CT), while metastases of the N3 lymph node should be confirmed pathologically by biopsy.

PRD was evaluated by a multidisciplinary clinical team (MDT), including oncologists, radiological specialists and thoracic surgeons, and was defined as (1) tumour invading the root of the main vessels, trachea or other unresectable organs which affected indispensable physiological functions and (2) IIIB or more advanced NSCLC not benefiting from surgery alone, with definitive chemoradiation as the standard therapeutic regimen [13]. Surgical evaluation before and after neoadjuvant treatment is described in Additional file 2: Table S2.

### Therapeutic procedure

Patients received the following drugs intravenously in each 21-day treatment cycle: toripalimab (240 mg) on day 1, carboplatin (area under curve 5) on day 1, pemetrexed (500 mg/m^2^) on day 1 for adenocarcinoma (AD) and NSCLC-not otherwise specified (NOS), paclitaxel (175 mg/m^2^) on day 1 or gemcitabine (1000 mg/m^2^) on day 1 and day 8 for squamous cell carcinoma (SQ). After 2 cycles, radiographic re-evaluation was performed. If surgery was feasible, as evaluated by MDT, it was planned 28–42 days after the first day of the last treatment cycle; otherwise, additional cycles were considered. If surgery could not be realized after 4 treatment cycles, the therapeutic schedule was reformulated according to neoadjuvant therapeutic efficiency. Notably, complete resection was evaluated as being achieved in disease with N3 metastasis only when lymph node downstaging was confirmed pathologically. Adjuvant chemotherapy was conducted 4–6 weeks after surgery. Adjuvant immunotherapy was performed at the discretion of the patients and lasted 1 year (240mg, every 3 weeks).

### Safety and response evaluation

Adverse events and abnormal laboratory findings were monitored every week and graded according to National Cancer Institute Common Terminology Criteria for Adverse Events (CTCAE) version 5.0. Investigators discussed and determined whether adverse events were treatment related. Treatment was considered to be delayed or ceased if treatment-related adverse events (TRAEs) occurred and resumed if certain criteria were met. Dose reduction was permitted for chemotherapy drugs but not for toripalimab. Drug toxicity was monitored for 3 months after the last dose of neoadjuvant treatment. Surgical complications and mortality were monitored for 1 month after surgery.

Radiologic and pathologic responses were evaluated by specialists at the Department of Radiology and the Department of Pathology, Shanghai Pulmonary Hospital. Radiologic response was determined according to Response Evaluation Criteria for Solid Tumor (RECIST) version 1.1. Pathological response was assessed according to the protocol of Cottrell et al. [14]. Non-major pathological response (MPR) was defined as the presence of more than 10% viable tumour cells in the resected primary tumour, MPR was defined as the presence of 10% or less viable tumour cells in the resected primary tumour and complete pathological response (pCR) was defined as the absence of viable tumour cells in both the resected primary tumour and lymph nodes.

### Outcomes

The primary endpoints were safety and MPR, and the secondary endpoints were the rate of R0 resection, 5-year progression-free survival (PFS) and 5-year overall survival (OS). The exploratory endpoint was the identification of predictive biomarkers and transcriptomic characteristics of neoadjuvant chemoimmunotherapy. PFS was defined as the period from diagnosis to disease progression or death. OS was defined as the period from diagnosis to death. Postoperative follow-up was performed every 3 months during the first 2 years and every 6 months thereafter.

### RNA sequencing analysis

**Fig. 1:**
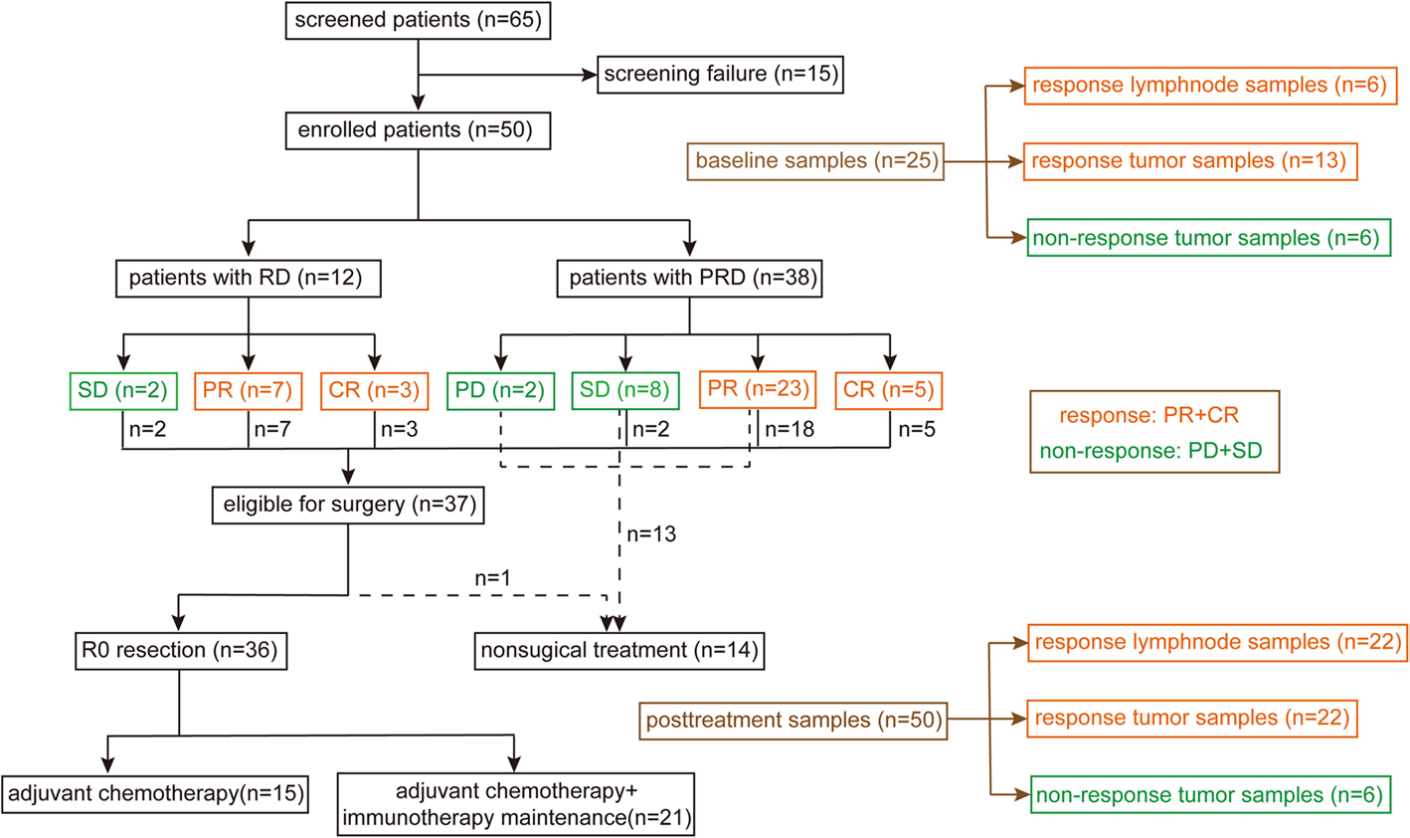
The flow chart of the enrolled patients and samples

In the trial, 25 baseline samples and 50 post-treatment samples were collected and successfully conducted for RNA sequencing analysis (Fig. 1). Total RNA from fresh frozen tissues was extracted with TRIzol. Sequencing libraries were generated using a NEBNext Ultra RNA Library Prep Kit for Illumina, and index codes were added to attribute sequences to each sample. The libraries were pooled, and paired-end sequencing (2×150 bp reads) was performed using an Illumina NovaSeq 6000. After RNA sequencing, raw sequencing data were trimmed using fastp and aligned to the GRCh38 reference genome by STAR with default settings [15, 16]. After obtaining BAM files, read counts were summarized by featureCounts, and TPMs (trans per million) were generated via Salmon [17, 18]. Batch effects were adjusted using the “combat” function in the sva package.

We used DESeq2, an R package, for differential expression analysis [19]. According to model gene count expression data, DESeq2 profile genes were used to calculate the log2-fold change, estimating the effect size and representing gene changes between comparison groups. Two-sided Wald test statistics were computed to examine differential expression across two sample groups. Genes with |Log2FoldChange| > 1 and Wald test *P* < 0.05 were defined as differentially expressed genes. Volcano plots were drawn to visualize the differential gene expression results.

For gene set enrichment analysis (GSEA), results for all protein-coding genes were ranked by fold change evaluated with the ‘GSEA’ algorithm [20]. ‘Hallmark’ and ‘KEGG’ gene sets were acquired from MSigDb. We filtered the GSEA results based on the criterion of *P* value < 0.05. We visualized candidate pathways based on the normalized enrichment score from the filtered list.

For tumour micro-environment estimation, the immune score of each sample was calculated via the ‘ESTIMATE’ R package [21]. Infiltration of immune cells was assessed via ‘mcpcounter’ [22]. For immune repertoire analysis, we employed the computational algorithm TRUST to evaluate the immune repertoire and to extract T and B cell receptor (TCR and BCR, respectively) complementarity-determining region 3 (CDR3) sequences [23, 24].

### Immunohistochemistry analyses

The formalin-fixed and paraffin-embedded tumour samples were obtained from 25 baseline patients and provided by the Pathology Department of Shanghai Pulmonary Hospital. Slides were stained with chitinase-3-like protein 1 (*CHI3L1*) antibody (1:800, CST, catalog No: 47066S) and were processed by standard procedures. Immunohistochemistry (IHC) score was calculated by the proportion of positive cells of tumour tissue (0–100%) by the average intensity of the positive staining (negative staining as 0, weak staining as 1, moderate staining as 2 and strong staining as 3), to obtain the score ranging from 0 to 300 for each sample.

### Statistical analyses

Drug safety, radiological response, PFS and OS were analysed in patients who received at least 1 cycle of neoadjuvant treatment. The R0 rate, pathological response and surgical outcomes were evaluated in patients who underwent surgical resection. A one-sample test based on an exponential distribution was applied to calculate the sample size. The sample size was set as 50 patients, providing 80% power to detect an MPR of 44% [25] compared to 20% for neoadjuvant chemotherapy [26], with a surgical rate of 60% and a two-sided type I error of 5%.

Continuous variables were compared using the Wilcoxon test, and the results are presented as the median and interquartile range (IQR). Categorical variables were compared using the Pearson chi-squared test or Fisher’s exact test, as appropriate, and the results were presented as frequencies and percentages. Factors between different groups divided by the radiological response and pathological response were analysed with a post hoc method. Exact two-sided 95% confidence intervals (CIs) were calculated with the Clopper–Pearson method. PFS and OS were assessed with the Kaplan–Meier method. The median follow-up time was calculated using the reverse Kaplan–Meier method. To evaluate the prognostic value of *CHI3L1*, we associated *CHI3L1* expression with OS and PFS, separately. In addition, both log-rank test and univariate cox regression were applied to confirm the prognostic value of *CHI3L1*. To separate patients into low- or high- *CHI3L1* groups, the cut-off was generated based on the association between *CHI3L1* expression and survival data using the survminer package. Reported *P* values were two-sided, with *P* < 0.05 considered statistically significant. The statistical analyses were performed in R (version 4.1.0).

### Data and code availability

We deposited the RNA-seq data in the Genome Sequence Archive database under accession number HRA002071. Codes utilized in this study are immediately available from the corresponding author upon reasonable request.

## Results

### Patient characteristics

**Fig. 2:**
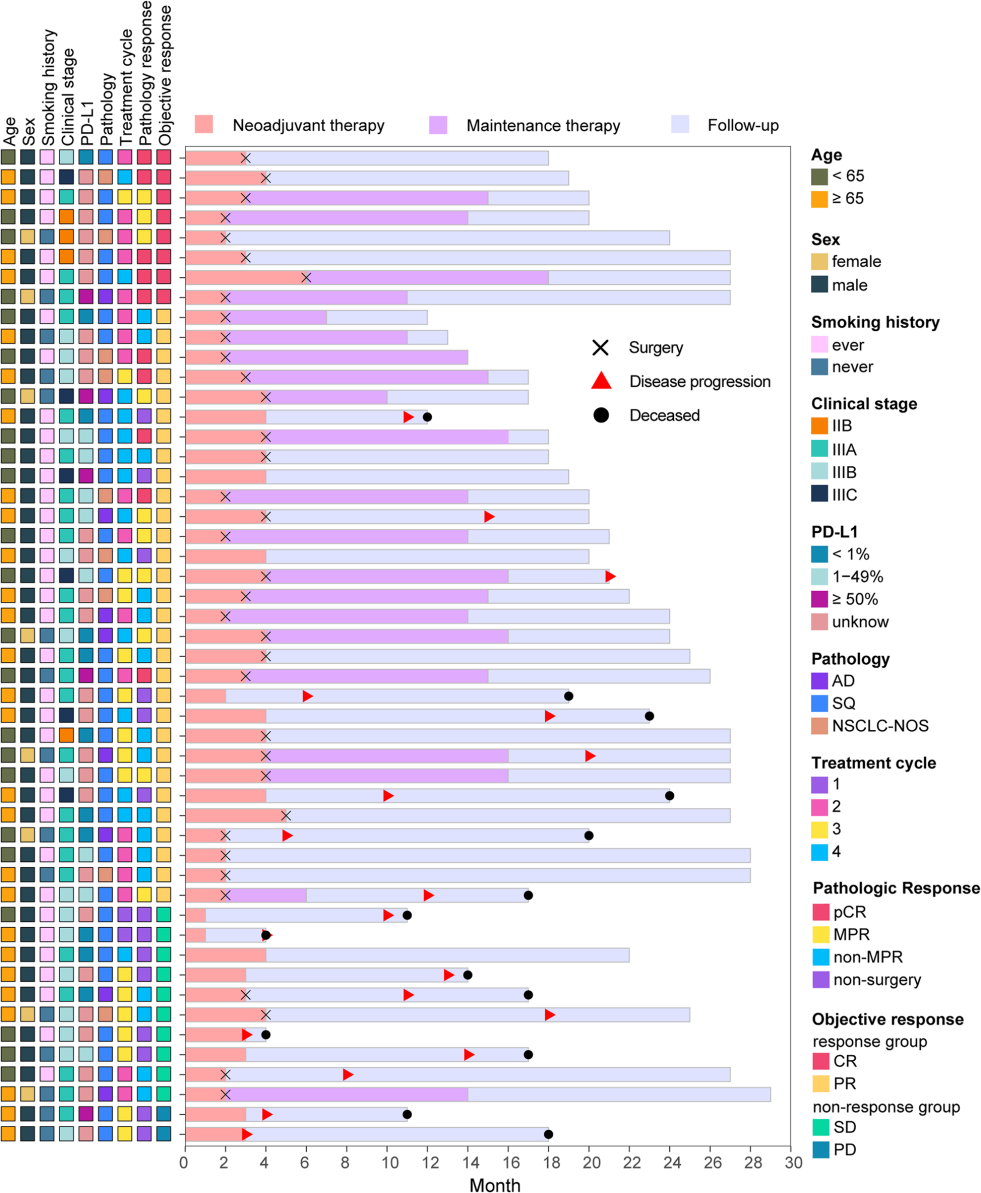
Swimming plot of clinical characteristics and follow-up

From June 1, 2020, to October 15, 2021, 50 eligible patients were enrolled at Shanghai Pulmonary Hospital (Fig. 1); the clinical characteristics of the patients are shown in Table 1. The median age was 66.0 (57.8–68.0) years, 42 (84.0%) patients were male and 35 (70.0%) had a smoking history. The disease distribution of stages IIB, IIIA, IIIB and IIIC consisted of 4 (8.0%), 24 (48.0%), 16 (32.0%) and 6 (12%) cases, respectively. Histopathological diagnosis from pre-treatment biopsies identified 32 (64.0%) patients with SQ, 9 (18.0%) with AD and 9 (18.0%) with NSCLC-not otherwise specified (NOS) (Fig. 2).

**Table 1:** Baseline characteristics

Characteristic		*N* = 50
Age (years)	Median (IQR)	66.0 (57.8–68.0)
Sex	Male	42 (84.0%)
	Female	8 (16.0%)
Smoking history	Ever	35 (70.0%)
	Never	15 (30.0%)
Stage	IIB	4 (8.0%)
	IIIA	24 (48.0%)
	IIIB	16 (32.0%)
	IIIC	6 (12.0%)
PD-L1 expression	<1%	11 (22.0%)
	1–49%	8 (16.0%)
	>49%	5 (10.0%)
	Unknown	26 (52.0%)
Histopathology	Squamous cell carcinoma	32 (64.0%)
	Adenocarcinoma	9 (18.0%)
	NSCLC-NOS	9 (18.0%)
Treatment cycle	1	2 (4.0%)
	2	18 (36.0%)
	3	15 (30.0%)
	4	15 (30.0%)

*IQR* interquartile range, *NSCLC-NOS* non-small-cell lung cancer-not otherwise specified

Before neoadjuvant therapy, 12 (24.0%) patients were evaluated to have RD and 38 (76.0%) to have PRD. According to the protocol, 2 (4.0%) patients received only 1 treatment cycle due to severe TRAEs; 18 (36.0%) received 2 treatment cycles and all underwent surgery. Fifteen patients (30.0%) received 3 treatment cycles; 1 had severe TRAEs, 2 with SD and 2 with PD did not receive an additional treatment cycle; and 15 (30.0%) received 4 treatment cycles (Additional file 3: Fig. S1a).

### Drug safety

The TRAEs that occurred are shown in Table 2, as recorded in 48 (96.0%) cases. TRAEs of grades 1–2 occurred in 31 (62.0%) patients and TRAEs of grades 3–5 in 17 (34.0%). The most common grade 1 or 2 TRAEs were neutropenia (16, 32.0%), rash (16, 32.0%) and anaemia (14, 28.0%). The most common grade 3 to 5 TRAEs were neutropenia (10, 20.0%), anaemia (4, 8.0%) and thrombocytopaenia (4, 8.0%). Three (6.0%) patients exhibited severe TRAEs involving myelosuppression, drug-induced liver injury and death related to haemoptysis. A patient with drug-induced liver injury was diagnosed as having immunotherapy-related AEs by liver biopsies and recovered after corticosteroid treatment. The deceased patient was found to have a large necrotic cavity in the tumour lesion after receiving the first cycle of treatment and suffered from haemoptysis and concomitant pneumonia for 4 months. TRAEs leading to treatment delay occurred in 7 (14.0%) patients and treatment discontinuation occurred in 4 (8.0%) patients.

**Table 2:** Treatment-related adverse events

	Grades 1–2	Grades 3–5
Neutropaenia	16 (32.0%)	10 (20.0%)
Anaemia	14 (28.0%)	4 (8.0%)
Rash	16 (32.0%)	0
Hepatic dysfunction	10 (20.0%)	2 (4.0%)
Thrombocytopaenia	7 (14.0%)	4 (8.0%)
Nausea	13 (26.0%)	0
Pulmonary infection	3 (6.0%)	2 (4.0%)
Renal dysfunction	4 (8.0%)	1 (2.0%)
Thyroid dysfunction	1 (2.0%)	0
Pulmonary embolism	0	1 (2.0%)
Fever	2 (4.0%)	1 (1.0%)
Febrile neutropaenia	0	1 (2.0%)
Haemoptysis	2 (4.0%)	1 (2.0%)
Atrial fibrillation	2 (4.0%)	0
Paresthaesia	2 (4.0%)	0
Hiccup	1 (2.0%)	0
Diarrhoea	1 (2.0%)	0

### Surgical outcomes

After the last cycle of neoadjuvant therapy, 37 (74.0%) patients were evaluated to be eligible for surgery, including all 12 (100.0%) with RD and 25 (68.4%) out of 38 with PRD. The 3 patients with severe TRAEs were assessed to have PRD before neoadjuvant treatment and did not receive surgical resection. The reasons why the other patients did not receive surgical resection are shown in Additional file 2: Table S2.

Overall, 1 (2%) patient with PRD refused surgery owing to the potential risk; 36 (72.0%) patients received surgery, and R0 resection was achieved in all patients. Lobectomy was performed in 35 patients; 1 patient underwent sub-lobectomy due to insufficient pulmonary function. No patient experienced treatment-related surgery delay. VATS (video-assisted thoracic surgery) was performed in 18 (50.0%) patients; conversion to thoracotomy was achieved in 4 (11.1%) cases. The perioperative outcomes of the cohort are shown in Additional file 4: Table S3. VATS was associated with a lower drainage volume (*P*=0.013), reduced drainage time (*P*=0.007) and shorter postoperative stay (*P*=0.034). Perioperative complications occurred in 5 (13.9%) patients, including 2 (5.6%) cases of persistent air leakage, 1 (2.8%) pneumonia, 1 (2.8%) postoperative haemorrhage and 1 (2.8%) chylothorax in the thoracotomy group. No reoperation or death occurred.

### Efficacy of neoadjuvant therapy

According to RECIST 1.1 criteria, the objective response rate was 76.0% (38/50), consisting of 8 (16.0%) patients with complete response (CR) and 30 (60.0%) with partial response (PR). In addition, 10 (20.0%) patients had stable disease (SD), and 2 (4.0%) had progressive disease (PD). The treatment response for each patient in different treatment cycles is shown in Additional file 3: Fig. S1a. Of the 36 patients undergoing surgery, 23 (63.9%) achieved lymph node downstaging: N3 downstaging to N0 in 3 (8.3%), N2 downstaging to N1 in 2 (5.5%), N2 downstaging to N0 in 10 (27.8%) and N1 downstaging to N0 in 8 (22.2%). According to postoperative pathological evaluation, 20 (55.6%) patients achieved MPR, including 10 (27.8%) with pCR, as shown in Table 3. The patients’ demographic characteristics, including age, sex, smoking history, PD-L1 expression and preoperative histopathologic type, were similar, except for clinical stage (*P*=0.023) (Table 4). A significant correlation was identified between the radiological response and pathological response (correlation=0.56, *P*<0.01) (Additional file 3: Fig. S1b).

**Table 3:** Radiological response and pathological response

Radiological response (*N*=50)	
Complete response	8 (16.0%)
Partial response	30 (60.0%)
Stable disease	10 (20.0%)
Progressive disease	2 (4.0%)
Pathological response (*N*=36)	
Non-MPR	16 (44.4%)
MPR	20 (55.6%)
CPR	10 (27.8%)
Downstaging of nodal status (*N*=36)	
N3 to N0	3 (8.3%)
N2 to N1	2 (5.5%)
N2 to N0	10 (27.8%)
N1 to N0	8 (22.2%)

**Table 4:** Comparison of clinical characteristics between the MPR group and the non-MPR group

	Non-MPR	MPR	*P*
Patients	16 (44.4%)	20 (55.6%)	
Age	66.5 (62.0–68.0)	60.5 (54.5–67.8)	0.147
Sex			
Male	12 (75.0%)	16 (80.0%)	
Female	4 (25.0%)	4 (20.0%)	1.000
Smoking history			
No	6 (37.5%)	6 (30.0%)	
Yes	10 (62.5%)	14 (70.0%)	0.729
Treatment cycles			
2	8 (50.0%)	10 (50.0%)	
3	6 (37.5%)	4 (20.0%)	
4	2 (12.5%)	6 (30.0%)	0.321
Adverse effect			
No	0	2 (10.0%)	
Grades 1–2	13 (81.2%)	12 (60.0%)	
Grades 3–5	3 (18.8%)	6 (30.0%)	0.184
Pre-treatment PD-L1 expression			
Negative	6 (37.5%)	2 (10.0%)	
Positive	2 (12.5%)	8 (40.0%)	
Unknown	8 (50.0%)	6 (30.0%)	0.054
Pathology			
Squamous cell carcinoma	8 (50.0%)	11 (55.0%)	
Adenocarcinoma	5 (31.3%)	4 (20.0%)	
Non-small-cell lung cancer	3 (18.8%)	5 (25.0%)	0.724
Clinical stage			
IIB	1 (6.3%)	3 (15.0%)	
IIIA	13 (81.2%)	7 (35.0%)	
IIIB	2 (12.5%)	7 (35.0%)	
IIIC	0	3 (15.0%)	0.023
Radiological response			
CR	0	8 (40.0%)	
PR	12 (75.0%)	12 (60.0%)	
SD	4 (25.0%)	0	<0.001

*MPR* major pathologic response, *CR* complete response, *PR* partial response, *SD* stable disease, *PD-L1* programmed cell death ligand 1

### Survival

The median follow-up was 22.0 (95% CI: 22.0–27.0) months at the time of data cut-off (Oct 31, 2022). The median PFS and OS were not reached of the 50 enrolled patients, PFS was 76.0% (95% CI: 65.0–88.8%) and OS was 90.0% (95% CI: 82.1–98.7%) at 12 months. Of the patients who underwent surgery, 21 (58.3%) received adjuvant immunotherapy maintenance, disease progression occurred in 3 (14.3%) patients and death occurred in 1 (4.8%) patient. The PFS was 42.9% (95% CI: 23.4–78.5%) and OS was 64.3% (95% CI: 43.5–95.0%) at 12 months in the non-surgery subgroup; PFS was 88.9% (95% CI: 79.2–99.8%) and OS was 100.0% at 12 months in the surgery subgroup.

**Fig. 3:**
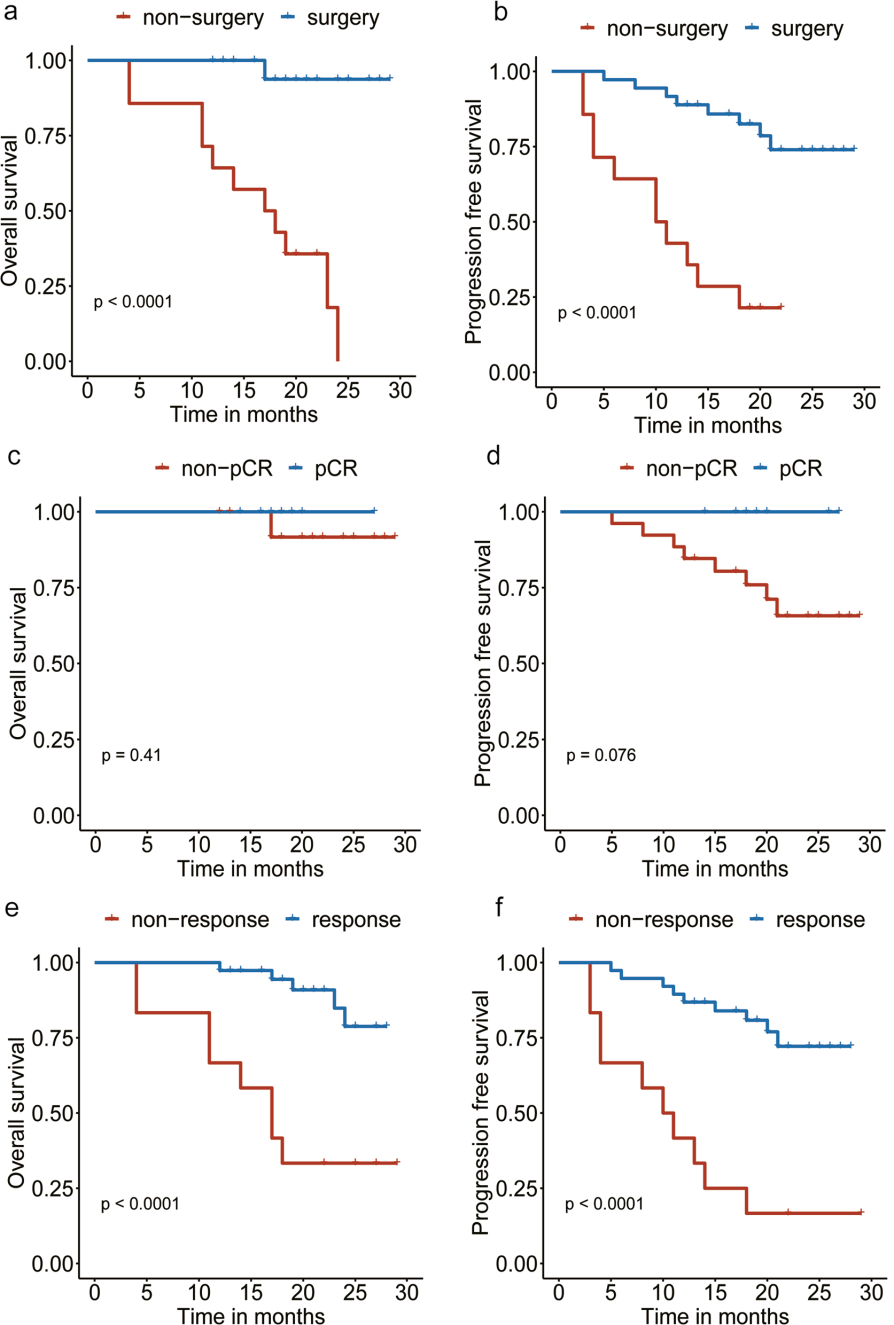
The survival analysis. **a** The difference in OS between the surgery group and the non-surgery group. **b** The difference in PFS between the surgery group and the non-surgery group. **c** The difference in OS between the pCR group and the non-pCR group. **d** The difference in PFS between the pCR group and the non-pCR group. **e** The difference in OS between the response group and the non-response group. **f** The difference in PFS between the response group and the non-response group

Thus, patients in the surgery group had a better PFS (*P*<0.001) and OS (*P*<0.001) than those in the non-surgery group (Fig. 3a, b). Patients with complete pathological response also showed better survival tendency (pCR vs. non-pCR) (Fig. 3c, d). In addition, the PFS (*P*<0.001) and OS (*P*<0.001) were significantly longer in the radiologic response group (CR+PR) than the non-response group (SD+PD) (Fig. 3e, f). Patients with immunotherapy maintenance had moderately longer PFS than those without immunotherapy maintenance, but no significant difference was observed (Additional file 3: Fig. S1c and d). There was no difference in PD-L1 subgroups (PD-L1 negative vs. PD-L1 positive) with regard to OS or PFS (Additional file 3: Fig. S1e and f).

### RNA sequence analysis

#### Differential expression between response and non-response groups at baseline

**Fig. 4:**
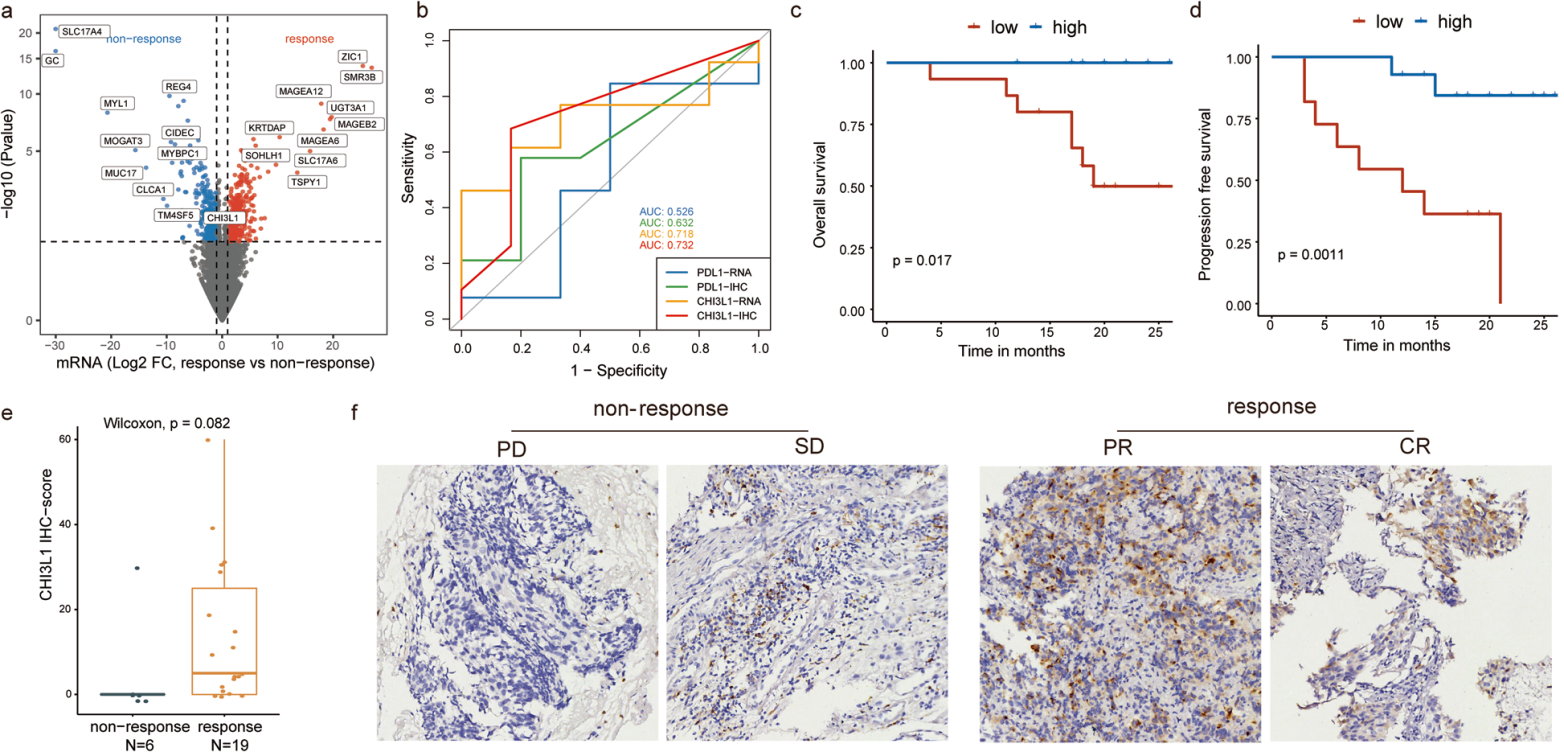
The differential expression analysis of baseline tumour samples. **a** Differential expression between the response group and the non-response group in baseline tumour samples. **b** *CHI3L1* expression in baseline tumour samples showed a better predictive ability to treatment response than PD-L1. **c** The difference in OS between high and low *CHI3L1* IHC scores in baseline tumour samples. **d** The difference in PFS between high and low *CHI3L1* IHC scores in baseline tumour samples. **e** *CHI3L1* IHC expression comparison in baseline tumour samples between non-response and response groups. **f** Representative IHC images for *CHI3L1* in different response groups

At baseline, 868 genes were found to be differentially expressed (513 higher and 355 lower in the response group). *CHI3L1* was significantly over-represented in the response group (Fig. 4a).

Patients with higher *CHI3L1* RNA-seq expression also showed better PFS (*P*=0.003) (Additional file 5: Fig. S2a). Higher *CHI3L1* RNA-seq expression was enriched for antigen presentation and procession (Additional file 5: Fig. S2b), which might render patients with higher expression sensitive to immunotherapy.

Notably, *CHI3L1* expression at baseline showed a higher predictive ability to treatment response than PD-L1 in the IHC level or RNA level (Fig. 4b). Patients with higher *CHI3L1* IHC scores also showed better OS (*P*=0.017) and PFS (*P*=0.001) (Fig. 4c, d). Besides, baseline tumour in the response group showed a moderately higher *CHI3L1* IHC score (Fig. 4e). The representative IHC images for *CHI3L1* in different response groups are shown in Fig. 4f.

Furthermore, when validated in another immunotherapeutic dataset, *CHI3L1* expression was also significantly higher in the response group at baseline (Additional file 5: Fig. S2c), and baseline *CHI3L1* expression could predict immunotherapy efficiency in GSE91061 (Additional file 5: Fig. S2d) [27]. In the immunotherapy cohorts for NSCLC, GSE161537 and GSE135222, patients with higher *CHI3L1* expression showed better OS (*P*=0.0018) and PFS (*P*=0.004) (Additional file 5: Fig. S2e and f) [28, 29].

For the analysis of immune repertoire in the baseline tumour samples, we found that BCR clonality (AUC=0.769) and BCR reads (AUC=0.744) at baseline also exhibited good predictive ability with regard to treatment efficiency (Fig. S3g and h).

#### Differential expression between baseline and post-treatment samples

**Fig. 5:**
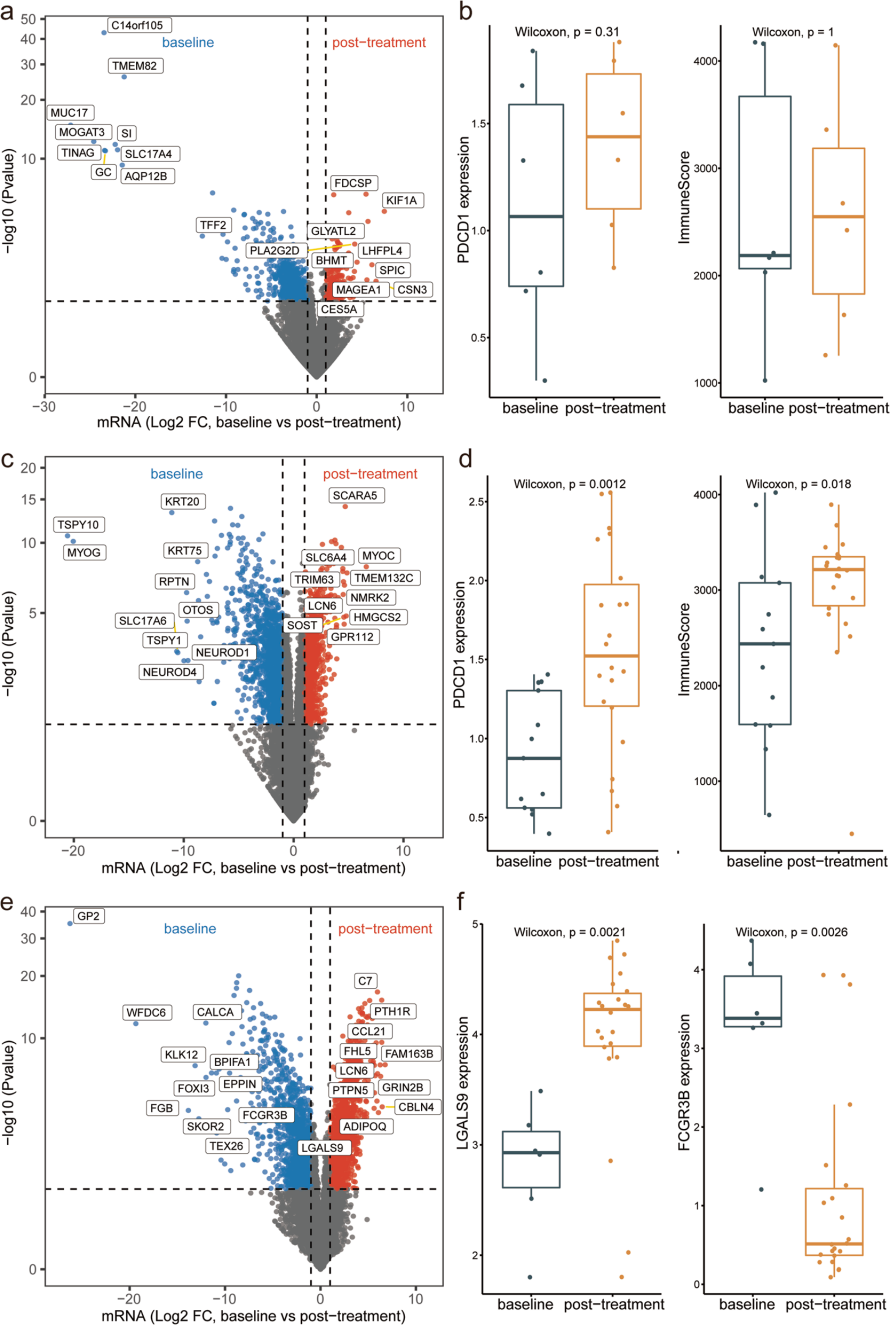
The differential expression analysis between baseline and post-treatment samples. **a** Differential expression between baseline and post-treatment tumour samples in the non-response group. **b** Expression of PD-1 and immune scores in baseline and post-treatment tumour samples in the non-response group. **c** Differential expression between baseline and post-treatment tumour samples in the response group. **d** Expression of PD-1 and immune scores in baseline and post-treatment tumour samples in the response group. **e** Differential expression between baseline and post-treatment lymph node samples in the response group. **f** LGALS9 and FCGR3B expression differences between baseline and post-treatment lymph node samples in the response group

When comparing baseline and post-treatment tumour samples, 727 genes were found to be differentially expressed in the non-response group (187 higher and 540 lower after treatment) (Fig. 5a). GSEA using hallmark and KEGG gene sets did not identify any immune-related pathways. Moreover, no difference was observed between baseline and post-treatment samples in terms of some immune checkpoints, such as *PD-1*, and immune scores also showed no significant change after immunotherapy treatment (Fig. 5b).

In the response group, 2360 genes were found to be differentially expressed between baseline and post-treatment tumour samples (1001 higher and 1359 lower after treatment) (Fig. 5c). GSEA identified several immune-related processes, including ‘B cell receptor signalling’ and ‘antigen processing and presentation’, enriched specifically in the response group after treatment (Additional file 6: Fig. S3a). *PD-1* expression and the immune scores showed an increasing trend after treatment (Fig. 5d). T cell counts, especially CD8+ T cells, were also significantly higher after treatment (Additional file 6: Fig. S3b).

In the analysis of differential expression between baseline and post-treatment lymph node samples in the response group, 3222 genes were revealed (1555 higher and 1667 lower after treatment) (Fig. 5e). Some immune checkpoints, such as *LGALS9*, displayed an increasing trend after treatment (Fig. 5f). However, neutrophils showed a decreasing tendency along with down-regulated *FCGR3B* expression (Additional file 6: Fig. S3c). In addition, neutrophil-related gene set was significantly down-regulated by GSEA analysis (Additional file 6: Fig. S3d) [30].

#### Differential expression between response and non-response groups after treatment

After treatment, 3127 genes were found to be differentially expressed between response and non-response tumour samples (1913 higher and 1214 lower in the response group) (Additional file 7: Fig. S4a). The transcript levels in the response group included immune checkpoints such as *HAVCR2* and *SIGLEC9* (Additional file 7: Fig. S4b), suggesting that chemoimmunotherapy might invoke feedback inhibition. In addition, we observed that several MHC family members, such as *HLA-DRA*, *HLA-DRB1*, *HLA-DPA1* and *HLA-DPB1* (Additional file 7: Fig. S4c), were highly expressed in response tumour samples. GSEA identified immune-related processes, including ‘antigen processing and presentation’, highly expressed in the response group. Conversely, gene sets for ‘estrogen response early’ and ‘estrogen response late’ were highly expressed in the non-response group (Additional file 7: Fig. S4d).

## Discussion

Trials of neoadjuvant chemoimmunotherapy for NSCLC with PRD and analysis of the molecular characteristics are limited to date. Here, we report the safety and effectiveness of neoadjuvant chemoimmunotherapy for patients with stage II–III NSCLC, including 12 patients with RD and 38 with PRD. R0 resection was achieved in 24 (63.2%) patients of PRD and 12 (100%) of RD with manageable TRAEs. Our trial obtained a 76.0% ORR and 55.6% (20/36) MPR, including 27.7% (10/36) pCR. With a median follow-up of 22 months, OS was 90.0% and PFS 76.0% at 12 months for all enrolled patients. In addition, several molecular characteristics of treatment efficiency were described based on RNA sequencing analysis.

Previous trials of neoadjuvant chemoimmunotherapy focused mainly on resectable IB-IIIA NSCLC [7, 31, 32]. In a retrospective study, 31 (60.8%) patients with initially unresectable NSCLC were reported to undergo surgery successfully after neoadjuvant immunochemotherapy. The high rate of conversion to surgery and the significant improvement of survival suggested the feasibility of neoadjuvant chemoimmunotherapy in unresectable NSCLC [9]. Nevertheless, prospective trials of neoadjuvant chemoimmunotherapy for initially unresectable NSCLC are still limited. Long et al. [33] investigated 13 (39.4%) cases of stage T3-4N2M0, and their promising results supported the further investigation. Of the 38 patients with PRD that was unresectable initially in our trial, 25 (65.7%) were eligible for surgery upon re-evaluation, and 24 (63.2%) eventually underwent surgery. Moreover, R0 resection was achieved in all patients. The high proportion of conversion and R0 resection rate preliminarily demonstrate the feasibility of neoadjuvant chemoimmunotherapy for NSCLC with PRD, which should be verified in the therapeutic and survival analyses.

The therapeutic effect after neoadjuvant chemoimmunotherapy in resectable NSCLC had been described in previous trials, with an MPR of 57–63% [7, 31, 32]. Our trial showed a comparable MPR of 55.6% in the surgical subgroup. Regarding all enrolled patients in our trial, the ORR of 76% was also similar to that of the NADIM trial, with an ORR of 76%, and a trial of neoadjuvant atezolizumab plus chemotherapy, with an ORR of 63%. TRAEs were recorded in 96% of patients in our trial, and most were grades 1–2, identical to the above trials [7, 31]. Therefore, neoadjuvant toripalimab plus chemotherapy was safe and feasible in more advanced NSCLC, consistent with resectable IB-IIIA NSCLC.

Survival was a crucial concern in neoadjuvant chemoimmunotherapy. In the trial of atezolizumab without adjuvant immunotherapy, 36.7% of patients were reported to have disease progression at a median follow-up of 12.9 months [7]. In the NADIM trial, patients received neoadjuvant chemoimmunotherapy followed by adjuvant immunotherapy maintenance for 1 year, and the PFS was 95.7% at 12 months. The OS and PFS rates of all patients in our trial were 92.7% and 75.6% at 12 months, respectively, with a median follow-up of 22.0 months. Hence, survival was higher than that of the atezolizumab trial but lower than the NADIM trial, for the difference of adjuvant therapy and stage distribution. In our trial, PFS in the immunotherapy maintenance cohort was also moderately longer, suggesting the effectiveness of adjuvant immunotherapy. The IMpower 010 trial also proved that adjuvant immunotherapy could improve the prognosis [34]. Therefore, the benefit of neoadjuvant chemoimmunotherapy and immunotherapy maintenance might reformulate the therapeutic regimen for NSCLC.

At present, predictive factors of neoadjuvant chemoimmunotherapy still need further investigation. We found that baseline PD-L1 expression, TRAEs and pathological diagnosis were not significantly different between MPR and non-MPR subgroups. In accordance with the atezolizumab trial, the PD-L1 expression could not predict OS and PFS in our trial, but was proved to be a prognostic predictor in the CheckMate 816 trial [7, 8]. No definitive predictors of therapeutic efficacy have been identified, and more sequencing-based studies are needed to identify the intrinsic and extrinsic molecular changes associated with neoadjuvant chemoimmunotherapy.

Regarding potential predictive markers for immunotherapy response, baseline *CHI3L1* expression showed better predictive ability compared with PD-L1 in our trial. *CHI3L1* was originally discovered in mouse breast cancer cells [35]. It was known to be expressed by a variety of immune cells and stimulated by mediators including IL-13, IL-6, IL-1β and IFN-γ [36, 37]. A recent study reported that *CHI3L1* regulated PD-L1 and anti-*CHI3L1*-PD-1 antibodies to elicit synergistic antitumour responses, reflecting that *CHI3L1* might constitute a target to augment the efficacy of anti-PD-1 in lung cancer [38]. In addition, GSEA analysis revealed that antigen presentation and procession pathway was enriched in the higher *CHI3L1* expression group, which might render patients with higher *CHI3L1* expression sensitive to immunotherapy. We also validated the predictive value of *CHI3L1* in other immunotherapy-treated datasets for NSCLC. Overall, *CHI3L1* was a promising predictive marker for immunotherapy efficiency.

In the analysis of baseline and post-treatment tumour samples, *PD-1* expression and immune scores showed an increasing trend after chemoimmunotherapy from the response group, reflecting remodelling of the tumour micro-environment (TME) after chemoimmunotherapy. However, in the non-response group, no significant TME changes, such as PD-1 expression and immune scores, were observed. In response lymph node samples, neutrophils exhibited a decreasing tendency after chemoimmunotherapy along with down-regulated *FCGR3B* expression. Neutrophils were emerging as potential biomarkers related to the immune context in patients with cancer [39]. The neutrophil population was heterogeneous, with both pro- and antitumour properties. In general, neutrophils were associated with cancer progression, and a key subpopulation, known as immature neutrophils, had a potentially detrimental impact [40]. Immature neutrophils might be a key subpopulation of immunotherapy resistance, and decreasing neutrophil counts might enhance immunotherapy efficiency.

After treatment, response tumour samples showed ‘antigen presentation and processing’ to be over-represented. However, gene sets for ‘estrogen response early’ and ‘estrogen response late’ were highly expressed in the non-response group. Oestrogen had an important role in lung cancer carcinogenesis [41, 42], reported to adversely affect the prognosis of patients with lung cancer [43]. Oestrogen decreased *IFN-γ* and *IL-2* expression in T cells co-cultured with tumour cells, suggesting E2-induced inhibition of T cell function [44]. The suppressive action of T cells was dependent on *PD-1* expression in Tregs, which was also increased by oestrogen [45]. Despite significant clinical success for immune checkpoint blockade therapies in the treatment of certain cancers, partial response rates and acquired resistance necessitated the development of strategies to boost immunotherapeutic responses. The data summarized herein pointed to the oestrogen pathway as a regulator of immune responses, suggesting that a clinical benefit might be derived from combining oestrogen-blocking agents with immune checkpoint inhibitors, especially in immunotherapy-resistant patients.

This trial had several limitations. First, it was a single-arm trial with relatively small populations, and patients eligible for surgery after neoadjuvant therapy should be randomized into a surgical group and a non-surgery group in further trials. Second, the cases were heterogeneous, including 12 (24.0%) patients with RD and 38 (76.0%) with PRD. Additionally, lymph node diagnosis was not confirmed pathologically in all cases, which might affect the accuracy of the clinical stage, and the survival after neoadjuvant chemoimmunotherapy should be evaluated in a longer follow-up period.

## Conclusions

Neoadjuvant toripalimab plus chemotherapy is safe and effective with high MPR and manageable TRAEs in patients with stage II–III NSCLC, which even converts a high proportion of PRD into RD. We also describe the molecular characteristics of neoadjuvant chemoimmunotherapy in NSCLC and find *CHI3L1* to be a promising predictive marker for treatment efficiency.

## Supplementary Information

**Additional file 1: Table S1.** Inclusion and exclusion criteria.

**Additional file 2: Table S2.** Surgical evaluation before and after neoadjuvant therapy.

**Additional file 3: Figure S1**. (a) Treatment response for each patient in different treatment cycles (b) A significant correlation was identified between radiological response and pathological response. (c) The difference in OS between the adjuvant immunotherapy maintenance group and the non-maintenance group. (d) The difference in PFS between the adjuvant immunotherapy maintenance group and the non-maintenance group. (e) The difference in OS between the PD-L1 positive group and the negative group. (f) The difference in PFS between the PD-L1 positive group and the negative group.

**Additional file 4: Table S3.** Comparison of perioperative outcomes between the VATS group and the thoracotomy group.

**Additional file 5: Figure S2.** (a) The difference in PFS between high and low *CHI3L1* RNA expression in baseline tumor samples. (b) Higher *CHI3L1* RNA expression was enriched for antigen presentation and procession. (c) Higher *CHI3L1* expression in the response group at baseline. (d) *CHI3L1* expression in baseline samples could predict immunotherapy efficiency. (e) The difference in OS between high and low *CHI3L1* expression. (f) The difference in PFS between high and low *CHI3L1* expression. (g) BCR clonality at baseline exhibited a predictive ability to chemoimmunotherapy efficiency. (h) BCR reads at baseline exhibited predictive ability to chemoimmunotherapy efficiency.

**Additional file 6: Figure S3.** (a) Normalized enrichment scores for GSEA analysis between baseline and post-treatment tumor samples in response group. (b) Increasing trend of T cells after treatment in response tumor samples. (c) Neutrophils showed a decreasing tendency after treatment in response lymph node samples. (d) Neutrophils related gene set was significantly down-regulated after treatment in response lymph node samples.

**Additional file 7: Figure S4.** (a) Differential expression between response and non-response tumor group after treatment. (b) The HAVCR2 and SIGLEC9 expression in response and non-response tumor samples after treatment. (c) Higher expression of MHC family members in response tumor sample after treatment. (d) Normalized enrichment scores for GSEA analysis between post-treatment response and non-response group in tumor samples.

## Data Availability

RNA sequencing data was deposited in the Genome Sequence Archive database under accession number HRA002071. Data and codes utilized in this study are immediately available from the corresponding author upon reasonable request.

## Abbreviations

AD: Adenocarcinoma
*CHI3L1*: Chitinase-3-like protein 1
CI: Confidence interval
CR: Complete response
CT: Computed tomography
GSEA: Gene set enrichment analysis
IQR: Interquartile range
MDT: Multidisciplinary clinical team
MPR: Major pathological response
NSCLC: Non-small-cell lung cancer
ORR: Objective response rate
OS: Overall survival
pCR: Complete pathological response
PD: Progressive disease
PD-1: Programmed cell death receptor 1
PD-L1: Programmed cell death ligand 1
PET: Positron emission tomography
PFS: Progression-free survival
PR: Partial response
PRD: Potentially resectable disease
RD: Resectable disease
SD: Stable disease
SQ: Squamous cell carcinoma
TRAEs: Treatment-related adverse events
VATS: Video-assisted thoracic surgery
