# Supplementary material for: Safety and effectiveness of neoadjuvant PD-1 inhibitor (toripalimab) plus chemotherapy in stage II–III NSCLC (LungMate 002): an open-label, single-arm, phase 2 trial
Source: BMC Med. 2022 Dec 30;20:493. doi: 10.1186/s12916-022-02696-4 (PMC9801594; doi:10.1186/s12916-022-02696-4)
Supplement: Supplementary file 1 — Additional file 1: Table S1. Inclusion and exclusion criteria. [file 12916_2022_2696_MOESM1_ESM.docx]

**Additional file 1: Table S1.** Inclusion and exclusion criteria.

| Inclusion criteria:  1. Age ≥ 18 years.  2. Eastern Cooperative Oncology Group (ECOG) performance-status score of 0 or 1.  3. Histological or cytological diagnosis of non-small-cell lung cancer (NSCLC) and clinical stage Ⅱ-Ⅲ according to the TNM classification (American Joint Committee on Cancer 8th edition criteria) confirmed by radiological examination or EBUS.  4. Wild-type *EGFR* (epidermal growth factor receptor) and *ALK* (anaplastic lymphoma kinase).  5. At least 1 measurable lesion according to Response Evaluation Criteria for Solid Tumours (RECIST) version 1.1.  6. Life expectancy more than 12 weeks.  7. Adequate haematological function, liver function and renal function:  Haemoglobin greater than or equal to 9.0 g/dL (which can be maintained or exceeded by blood transfusion);  Red blood cell counts greater than or equal to 2.0×10^9/L;  Absolute neutrophil count (ANC) greater than or equal to 1.0×10^9/L;  Platelet count greater than or equal to 100×10^9/L;  Total bilirubin within the normal range;  Alanine glutamate transaminase, aspartate aminotransferase and alkaline phosphatase less than 2.5 times of the normal value;  Creatinine less than 1.5 times of the normal value; Creatinine clearance greater than or equal to 60 mL/min.  International standardized ratio of prothrombin time (INR) less than or equal to 1.5 and activated partial thrombin time (APTT) less than or equal to 1.5 times of the normal value in patients who had not received anticoagulation therapy. Patients receiving full or extra-gastrointestinal anticoagulant therapy were eligible as long as the dose of anticoagulant was stable for at least 2 weeks and the results of the coagulation test were within the normal range before enrolment in the study.  8. Without distant metastasis.  9. Complete resection expected.  10. Normal pulmonary function to tolerate surgery.  11. Women of child-bearing age must undergo a pregnancy test within 7 days before starting treatment with negative results. Patients of childbearing age should use condoms for contraception during the trial period and within 30 days at the end of the trial.  12. Signing informed consent. |
| --- |
| Exclusion criteria:  1. Previous antitumour treatment of NSCLC, including surgery, chemotherapy, local radiotherapy, targeted therapy or other exploratory therapy.  2. History of other tumours except NSCLC within 5 years.  3. Any unstable systemic comorbidity (uncontrolled hypertension, severe arrhythmia, etc.)  4. Any active, diagnosed or suspected autoimmune disease, any corticosteroid treatment.  5. Allergy to any component of toripalimab.  6. Symptomatic grade 3 or 4 interstitial lung disease.  7. Human immunodeficiency virus (HIV) infection.  8. Underwent major surgery of other systems or severe trauma within 2 months.  9. Pregnancy or breast-feeding.  10. Malabsorption.  11. Neurological or psychiatric disorders history including epilepsy or dementia and lack of treatment compliance.  12. Other situations in which the investigators thought the patients should not be included. |
