# Supplementary material for: Safety and effectiveness of neoadjuvant PD-1 inhibitor (toripalimab) plus chemotherapy in stage II–III NSCLC (LungMate 002): an open-label, single-arm, phase 2 trial
Source: BMC Med. 2022 Dec 30;20:493. doi: 10.1186/s12916-022-02696-4 (PMC9801594; doi:10.1186/s12916-022-02696-4)
Supplement: Supplementary file 2 — Additional file 2: Table S2. Surgical evaluation before and after neoadjuvant therapy. [file 12916_2022_2696_MOESM2_ESM.docx]

**Additional file 2: Table S2.** Surgical evaluation before and after neoadjuvant therapy.

| No | Before treatment | After last treatment cycle |
| --- | --- | --- |
| 1 | Left upper lobectomy with systematic mediastinal lymphadenectomy. | VATS Left upper lobectomy with systematic mediastinal lymphadenectomy. |
| 2 | Left lower lobectomy with systematic mediastinal lymphadenectomy. | VATS Left lower lobectomy with systematic mediastinal lymphadenectomy. |
| 3 | Lymph-node 4R enlarged greatly and could not be resected completely. | VATS right upper lobectomy with systematic mediastinal lymphadenectomy. |
| 4 | Left lower lobectomy with systematic mediastinal lymphadenectomy. | VATS Left lower lobectomy with systematic mediastinal lymphadenectomy. |
| 5 | Lymph-node 4L enlarged greatly; could not be resected completely. | VATS Left upper lobectomy with systematic mediastinal lymphadenectomy. |
| 6 | Right hilar lymph-nodes compressed the root of right pulmonary artery; mass compressed right inferior pulmonary vein; could not be resected completely. | Right lower lobectomy with systematic mediastinal lymphadenectomy. |
| 7 | Mass invaded right hilum and right lower lobe severely; could not be resected completely. | Right upper lobectomy with systematic mediastinal lymphadenectomy. |
| 8 | Mass invaded the root of pulmonary artery; could not be resected completely. | Right upper lobe, middle lobe and superior segment of right lower lobe resection with systematic mediastinal lymphadenectomy. |
| 9 | Metastasis of supraclavicular lymph-node; could not be resected completely. | Could not be resected completely. |
| 10 | Lymph-node 7 enlarged greatly and could not be resected completely. | Right lower lobectomy with systematic mediastinal lymphadenectomy. |
| 11 | Mass invaded the root of pulmonary artery; could not be resected completely. | Pericardiotomy and left upper lobectomy with systematic mediastinal lymphadenectomy. |
| 12 | Mass invaded the root of pulmonary artery; could not be resected completely. | Left upper lobectomy with systematic mediastinal lymphadenectomy. |
| 13 | Mass invaded aortic arch and the root of pulmonary artery; could not be resected completely. | Left upper lobectomy with systematic mediastinal lymphadenectomy. |
| 14 | Mass invaded the root of pulmonary vein; could not be resected completely. | Could not be resected completely. |
| 15 | Lymph-nodes 5L compressed the root of pulmonary artery; mass invaded the root of right pulmonary artery; could not be resected completely. | Could not be resected completely. |
| 16 | Right upper lobe sleeve resection with systematic mediastinal lymphadenectomy. | Right upper lobectomy with systematic mediastinal lymphadenectomy. |
| 17 | Metastasis of contralateral lymph-node 4L; could not be resected completely. | Could not be resected completely. |
| 18 | Mass invaded the root of main bronchus and the root of right pulmonary artery; could not be resected completely. | Could not be resected completely. |
| 19 | Mass invaded left lower lobe and the root of pulmonary vein; could not be resected completely. | Could not be resected completely. |
| 20 | Mass invaded left lower lobe; could not be resected completely. | Left upper lobectomy with systematic mediastinal lymphadenectomy. |
| 21 | Right hilar lymph-nodes compressed the root of pulmonary artery; could not be resected completely. | VATS right upper lobectomy with systematic mediastinal lymphadenectomy. |
| 22 | Mass invaded the root of pulmonary vein; could not be resected completely. | Right lower lobectomy with systematic mediastinal lymphadenectomy. |
| 23 | Right upper lobectomy with systematic mediastinal lymphadenectomy. | Right upper lobectomy with systematic mediastinal lymphadenectomy. |
| 24 | Lymph-node 4R enlarged greatly; mass invaded pulmonary artery, could not be resected completely. | VATS right upper lobectomy with systematic mediastinal lymphadenectomy. |
| 25 | Left upper lobectomy with systematic mediastinal lymphadenectomy. | Left upper lobectomy with systematic mediastinal lymphadenectomy. |
| 26 | Right upper lobectomy with systematic mediastinal lymphadenectomy. | VATS right upper lobectomy with systematic mediastinal lymphadenectomy; conversion to thoracotomy. |
| 27 | Multiple mediastinal lymph-node enlarged; could not be resected completely. | Could not be resected completely. |
| 28 | Right lower lobectomy with systematic mediastinal lymphadenectomy. | Right lower lobectomy with systematic mediastinal lymphadenectomy. |
| 29 | Mass invaded the root of main bronchus and right pulmonary artery; mediastinal lymph-node enlarged greatly; could not be resected completely. | Could not be resected completely. |
| 30 | Mass invaded the root of main bronchus and right pulmonary artery; could not be resected completely. | Could not be resected completely. |
| 31 | Metastasis of contralateral lymph-node 4R; could not be resected completely. | Left upper lobe wedge resection and left lower lobectomy with systematic mediastinal lymphadenectomy. |
| 32 | Mass invaded the root of pulmonary artery; could not be resected completely. | Could not be resected completely. |
| 33 | Right middle and lower lobectomy with systematic mediastinal lymphadenectomy. | Right middle and lower lobectomy with systematic mediastinal lymphadenectomy. |
| 34 | Right upper lobectomy with systematic mediastinal lymphadenectomy. | VATS right upper lobectomy with systematic mediastinal lymphadenectomy. |
| 35 | Lymph-node 5L compressed root of pulmonary artery; could not be resected completely. | Left upper lobectomy with systematic mediastinal lymphadenectomy. |
| 36 | Mass invaded main bronchus and pulmonary artery; could not be resected completely. | Severe adverse effect (death due to haemoptysis 4 months after treatment); could not be resected completely. |
| 37 | Lymph-node 7 enlarged greatly; could not be resected completely. | Severe adverse effect (drug-induced liver injury); could not be resected completely. |
| 38 | Left upper lobectomy with systematic mediastinal lymphadenectomy, poor pulmonary function. | VATS left upper lobe wedge resection. |
| 39 | Right upper lobectomy and right middle lobe wedge resection with systematic mediastinal lymphadenectomy. | VATS right upper lobectomy and right middle lobe wedge resection with systematic mediastinal lymphadenectomy. |
| 40 | Metastasis of supraclavicular lymph-node; mass invaded the root of right main bronchus; could not be resected completely. | Mass invaded the root of right main bronchus; could not be resected completely. |
| 41 | Metastasis of contralateral lymph-node 4R; could not be resected completely. | VATS left upper lobectomy with systematic mediastinal lymphadenectomy. |
| 42 | Mass invaded root of main bronchus; could not be resected completely. | VATS left lower lobectomy with systematic mediastinal lymphadenectomy. |
| 43 | Mass invaded the root of main bronchus and right pulmonary artery; lymph-node 7 enlarged greatly; could not be resected completely. | VATS right upper lobe sleeve resection with systematic mediastinal lymphadenectomy; conversion to thoracotomy. |
| 44 | Mass invaded the root of main bronchus and right pulmonary artery; could not be resected completely. | Right upper lobe sleeve resection with systematic mediastinal lymphadenectomy. |
| 45 | Mass invaded the root of pulmonary artery; could not be resected completely. | Right upper lobe double sleeve resection with systematic mediastinal lymphadenectomy, patient refused surgery for the potential surgical risk. |
| 46 | Metastasis of supraclavicular lymph-node; could not be resected completely. | VATS right upper lobectomy with systematic mediastinal lymphadenectomy. |
| 47 | Mass invaded root of left pulmonary artery; could not be resected completely. | VATS left lower lobectomy resection with systematic mediastinal lymphadenectomy. |
| 48 | Mass invaded root of pulmonary artery; could not be resected completely. | RATS left upper lobectomy with systematic mediastinal lymphadenectomy; conversion to thoracotomy. |
| 49 | Mass invaded root of main bronchus; could not be resected completely. | Left lower lobe sleeve resection with systematic mediastinal lymphadenectomy. |
| 50 | Right lower lobectomy with systematic mediastinal lymphadenectomy | Right lower lobectomy with systematic mediastinal lymphadenectomy |
| VATS, video-assisted thoracoscopic surgery; RATS, robotic-assisted thoracic surgery. | | |
