# Supplementary figures and images for: Safety and effectiveness of neoadjuvant PD-1 inhibitor (toripalimab) plus chemotherapy in stage II–III NSCLC (LungMate 002): an open-label, single-arm, phase 2 trial
Source: BMC Med. 2022 Dec 30;20:493. doi: 10.1186/s12916-022-02696-4 (PMC9801594; doi:10.1186/s12916-022-02696-4)

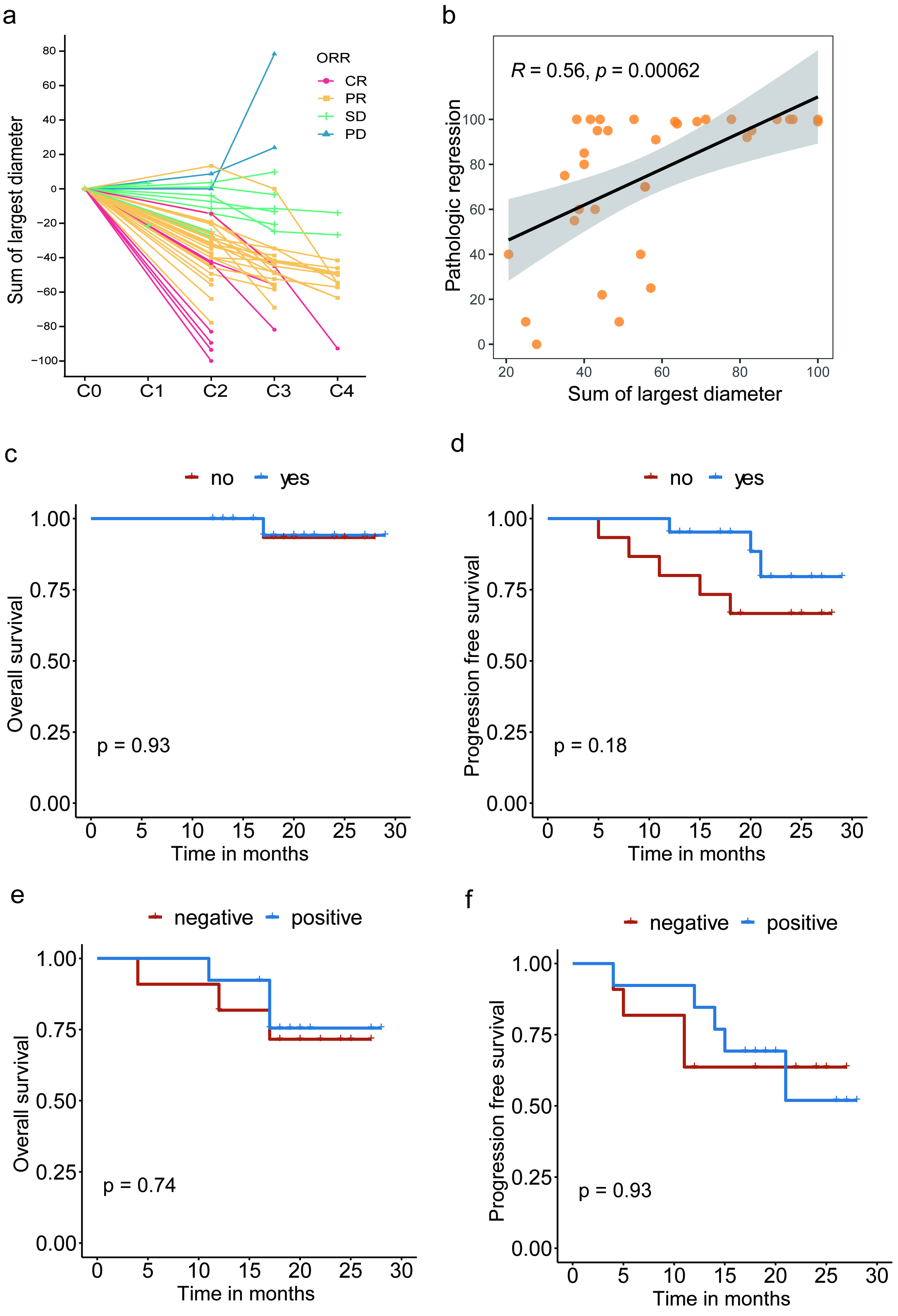

Supplement: Supplementary file 3 — Additional file 3: Figure S1. (a) Treatment response for each patient in different treatment cycles (b) A significant correlation was identified between radiological response and pathological response. (c) The difference in OS between the adjuvant immunotherapy maintenance group and the non-maintenance group. (d) The difference in PFS between the adjuvant immunotherapy maintenance group and the non-maintenance group. (e) The difference in OS between the PD-L1 positive group and the negative group. (f) The difference in PFS between the PD-L1 positive group and the negative group. [file 12916_2022_2696_MOESM3_ESM.tif]

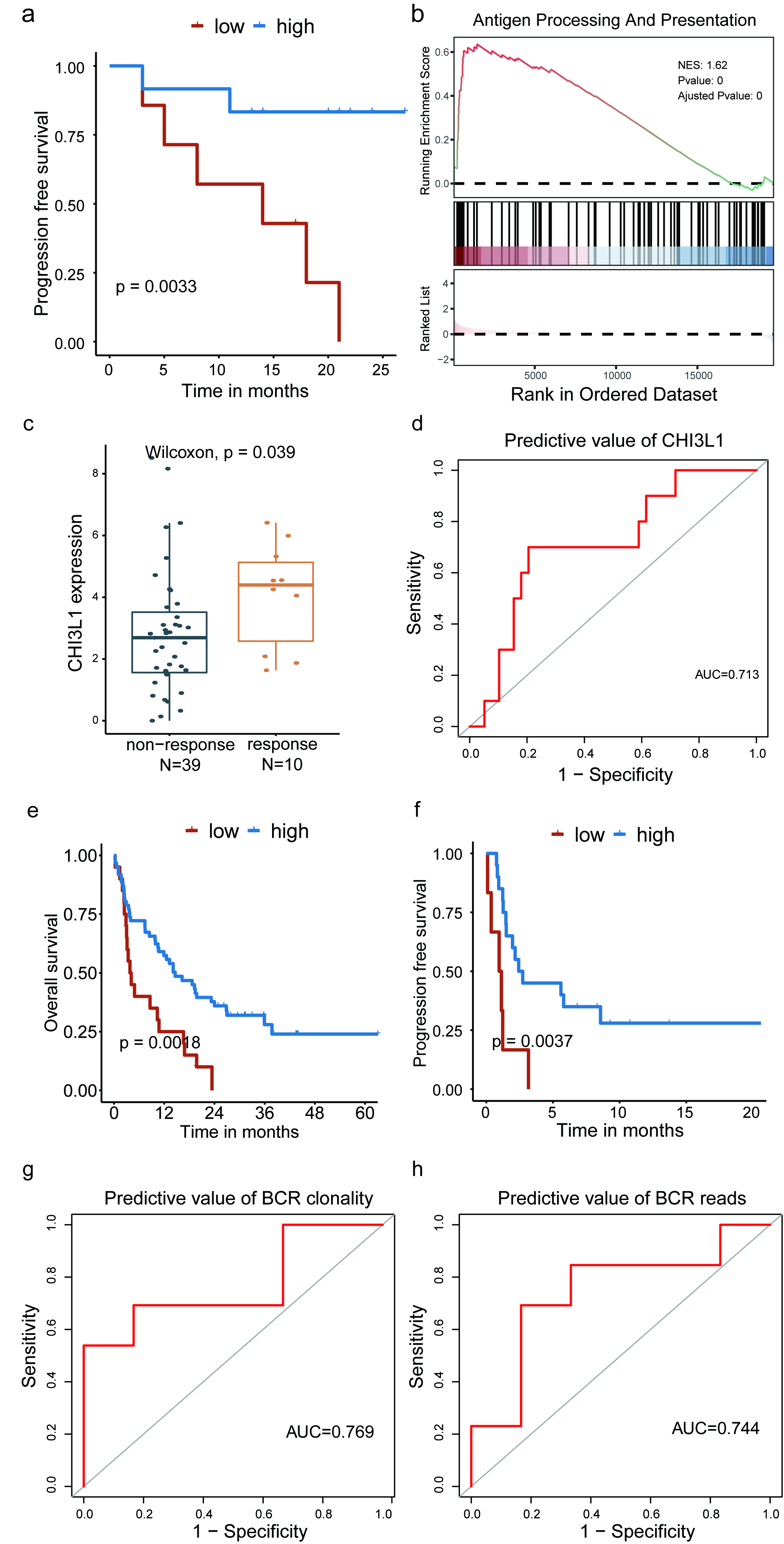

Supplement: Supplementary file 5 — Additional file 5: Figure S2. (a) The difference in PFS between high and low CHI3L1 RNA expression in baseline tumor samples. (b) Higher CHI3L1 RNA expression was enriched for antigen presentation and procession. (c) Higher CHI3L1 expression in the response group at baseline. (d) CHI3L1 expression in baseline samples could predict immunotherapy efficiency. (e) The difference in OS between high and low CHI3L1 expression. (f) The difference in PFS between high and low CHI3L1 expression. (g) BCR clonality at baseline exhibited a predictive ability to chemoimmunotherapy efficiency. (h) BCR reads at baseline exhibited predictive ability to chemoimmunotherapy efficiency. [file 12916_2022_2696_MOESM5_ESM.tif]

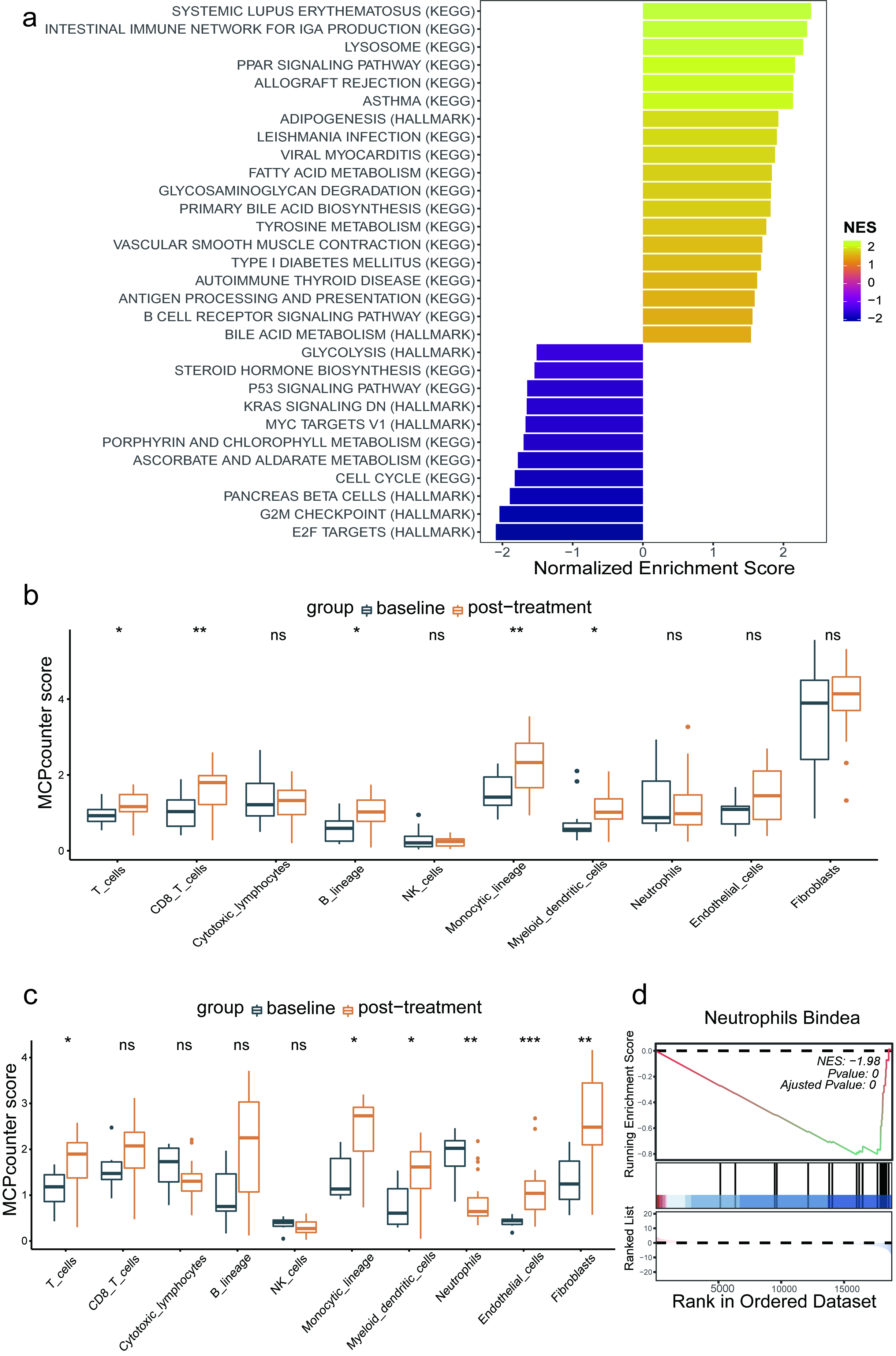

Supplement: Supplementary file 6 — Additional file 6: Figure S3. (a) Normalized enrichment scores for GSEA analysis between baseline and post-treatment tumor samples in response group. (b) Increasing trend of T cells after treatment in response tumor samples. (c) Neutrophils showed a decreasing tendency after treatment in response lymph node samples. (d) Neutrophils related gene set was significantly down-regulated after treatment in response lymph node samples. [file 12916_2022_2696_MOESM6_ESM.tif]

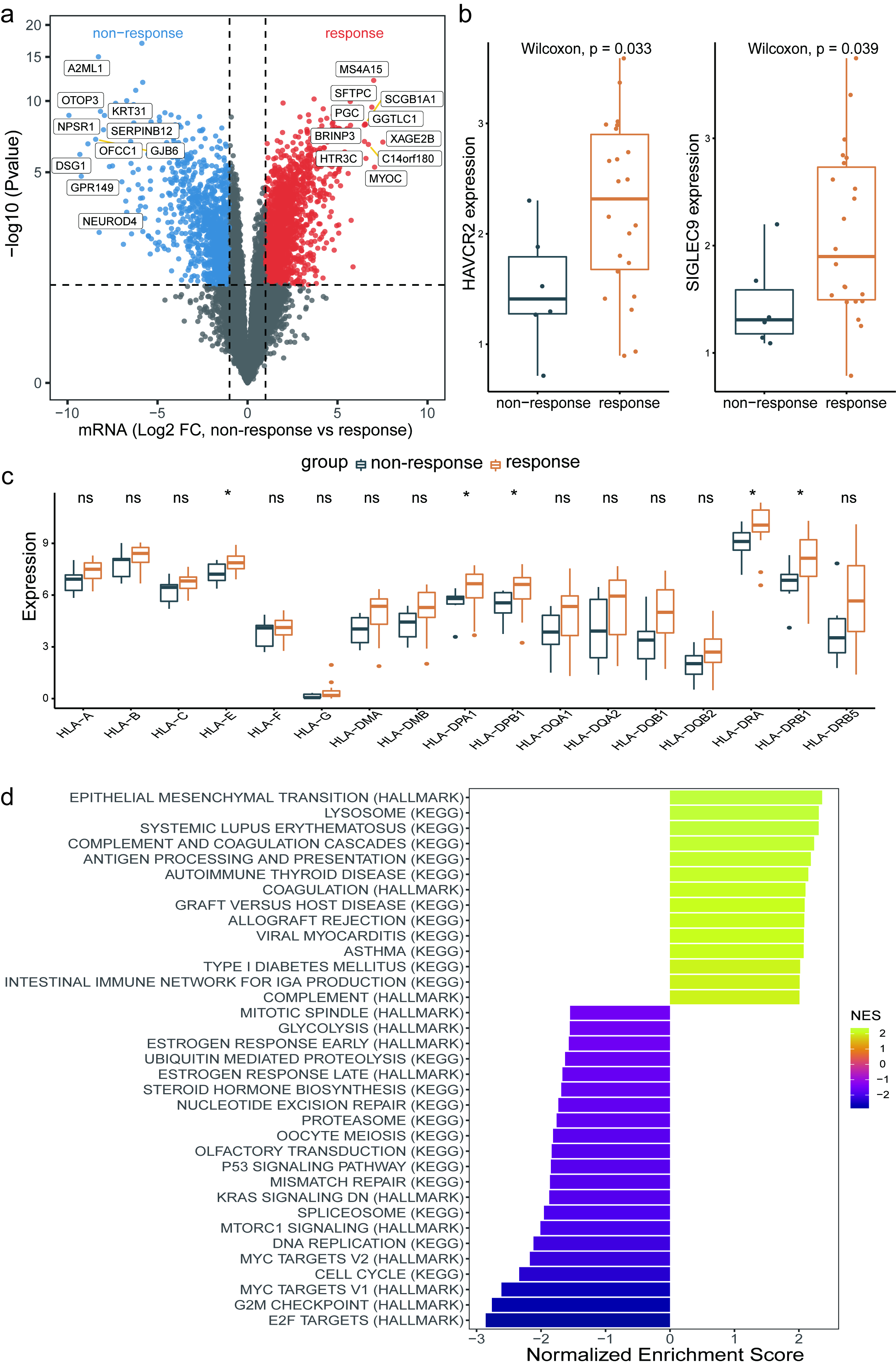

Supplement: Supplementary file 7 — Additional file 7: Figure S4. (a) Differential expression between response and non-response tumor group after treatment. (b) The HAVCR2 and SIGLEC9 expression in response and non-response tumor samples after treatment. (c) Higher expression of MHC family members in response tumor sample after treatment. (d) Normalized enrichment scores for GSEA analysis between post-treatment response and non-response group in tumor samples. [file 12916_2022_2696_MOESM7_ESM.tif]
