# Supplementary material for: Safety and effectiveness of neoadjuvant PD-1 inhibitor (toripalimab) plus chemotherapy in stage II–III NSCLC (LungMate 002): an open-label, single-arm, phase 2 trial
Source: BMC Med. 2022 Dec 30;20:493. doi: 10.1186/s12916-022-02696-4 (PMC9801594; doi:10.1186/s12916-022-02696-4)
Supplement: Supplementary file 4 — Additional file 4: Table S3. Comparison of perioperative outcomes between the VATS group and the thoracotomy group. [file 12916_2022_2696_MOESM4_ESM.docx]

**Additional file 4: Table S3.** Comparison of perioperative outcomes between the VATS group and the thoracotomy group.

|  | VATS | Thoracotomy | *P* |
| --- | --- | --- | --- |
| Patients | 18(50.0%) | 18(50.0%) |  |
| Sex (male) | 14 (77.8%) | 14 (77.8%) | 1.000 |
| Age (IQR) | 66.0(56.8-69.5) | 61.0(56.0-68.0) | 0.612 |
| Smoking history | 12(37.5%) | 12(73.3%) | 1.000 |
| Clinical stage (Ⅲ) | 16(88.9%) | 16(88.9%) | 1.000 |
| Blood loss(mL) | 50.0(50.0-100.0) | 75.0(50.0-125.0) | 0.160 |
| Operation time (min) | 143.5(120.0-185.5) | 180.0(147.0-216.0) | 0.074 |
| Drainage volume on day 1-3(mL) | 785.0 (595.0-986.2) | 1020.0(837.5-1352.5) | 0.013 |
| Drainage time(day) | 5.0(3.0-13.2) | 15.5(7.0-21.5) | 0.007 |
| Lymph node number | 14.0(10.5-20.2) | 14.0(9.8-18.2) | 0.987 |
| Lymph node station | 6.0(5.0-8.0) | 6.0(6.0-7.0) | 0.974 |
| Postoperative stay (day) | 5.0(4.0-6.0) | 6.0(5.0-7.2) | 0.034 |
| Complication |  |  | 0.045 |
| Pulmonary infection | 0 | 1(5.6%) |  |
| Air leakage | 0 | 2(11.1%) |  |
| Haemorrhage | 0 | 1(5.6%) |  |
| Chylothorax | 0 | 1(5.6%) |  |
| Conversion | 4(22.2%) | 0 |  |
| VATS, video-assisted thoracoscopic surgery; L, litre; mL, millilitre; min, minute. | | | |
